# Primary Percutaneous Coronary Intervention in a Patient with Haemophilia A

**DOI:** 10.1155/2013/189796

**Published:** 2013-12-19

**Authors:** S. Ashwin Reddy, Stephen P. Hoole, Martin W. Besser

**Affiliations:** Papworth Hospital NHS Foundation Trust, Papworth Everard, Cambridge CB23 3RE, UK

## Abstract

Haemophilia A is a rare genetic condition leading to coagulation factor VIII deficiency and thus predisposing to bleeding diathesis. Due to advances in treatment, life expectancy of haemophilia A sufferers is increasing, and the incidence and prevalence of coronary artery disease are rising. There have been many reported cases of acute myocardial infarction in such patients, who subsequently undergo elective percutaneous coronary intervention. We present the case of a 55-year-old gentleman presenting with an acute anterior full-thickness myocardial infarction who required emergency primary percutaneous coronary intervention.

## 1. Introduction

Haemophilia A is an X-linked recessive genetic defect, with a frequency of approximately 1 in 8,500 live births, resulting in coagulation factor VIII deficiency and bleeding diathesis. Haemorrhage into large joints and muscles, from the ears and nose, and following surgical challenge is a particular feature. The severity of the disease is determined by the factor VIII gene mutation, which in turn is reflected by the factor VIII level. Severe disease is defined as a factor VIII level of <1% normal, moderate disease 1–5%, and mild disease >5%. Since factor VIII is involved in the intrinsic clotting cascade, the APTT is prolonged. Platelet function, however, remains normal.

Haemophilia A is believed to have a direct protective effect in the development of coronary artery disease [1, 2]. Nevertheless, acute coronary syndrome can be provoked by the administration of recombinant factor VIII or DDAVP [3, 4]. Haemophilia A life expectancy in the developed world is almost normal, meaning that the incidence of coronary atherosclerosis and subsequent ischaemic heart disease in such patients is rising.

Primary percutaneous coronary intervention (PPCI) is the treatment of choice in those presenting with acute ST-segment elevation myocardial infarction (STEMI) but carries with it potential haemorrhagic risk due to the antithrombotics administered intraprocedurally. This risk is amplified in those with haemophilia A. We present the case of a patient with haemophilia A presenting with STEMI, not provoked by factor VIII replacement, who underwent successful PPCI.

## 2. Case Report

A 55-year-old gentleman was admitted via the primary PCI pathway after presenting with severe, persistent central chest pain following exertion. He had no prior history of coronary artery disease, and his risk factors included tablet-controlled type 2 diabetes mellitus and a current smoking history. His ECG demonstrated significant ST-segment elevation in leads V1-3, though he was clinically stable on arrival with no evidence of decompensating left ventricular failure.

The patient also reported a history of haemophilia A (codon 2164 mutation) with no prior major intra-articular or intramuscular haemorrhage. At presentation, he was well controlled on tranexamic acid maintenance therapy. Haematological advice was that he required recombinant factor VIII or desmopressin only prior to major surgery.

Given his lack of significant bleeding episodes and his clinical presentation with chest pain and ST-segment elevation, he was transferred for primary percutaneous coronary intervention without further delay.

**Figure 1:**
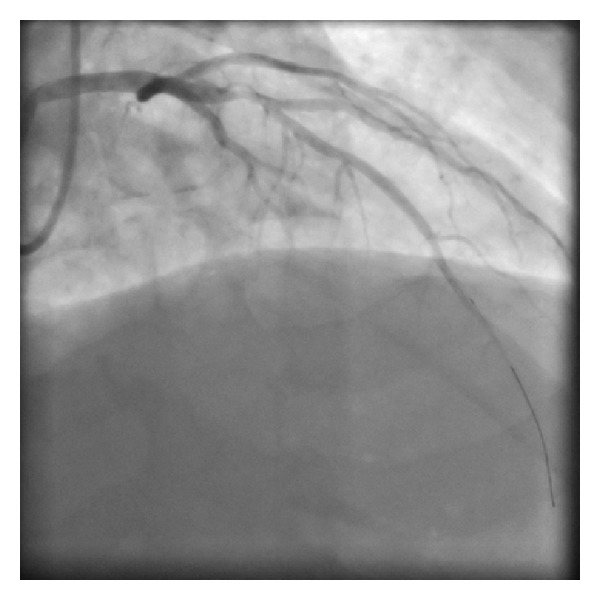
Tight proximal left anterior descending artery stenosis following wiring and attempted thrombus extraction.

**Figure 2:**
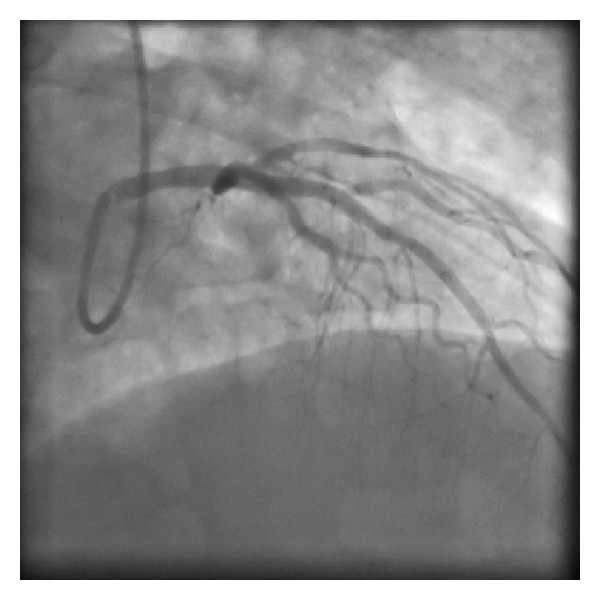
Good angiographic result with TIMI 3 flow following direct stenting of vessel.

Catheterisation was performed via the right radial artery using a 6F JR4 and XB 3.0 guide catheter. The patient was loaded with 300 mg aspirin and 60 mg prasugrel prior to the procedure and was then administered with 5000 units of heparin. A flow-limiting 95% stenosis in the proximal left anterior descending artery was identified, with no significant disease in the left mainstem, circumflex, or right coronary arteries (Figure 1). Two attempts at thrombus extraction using a Pronto LP device yielded very little, so the decision was made to direct stent the proximal LAD with a bare metal stent (3.5 × 23 mm Vision). The final result was excellent, with TIMI 3 flow down to the distal LAD (Figure 2). Haemostasis at the puncture site was achieved using a TR band filled with 13 mLs air.

After PCI, the patient was transferred to the High Dependency Unit for observation. His blood revealed an APTT of 54.1, factor VIII level of 60%, and a peak troponin I level of 6.72. He was well after procedure and haemodynamically stable, with no further chest pain and normalisation of his ST-segment elevation. There was no bleeding or haematoma formation at the right radial puncture site. No further haematological intervention was needed.

The patient had an unremarkable recovery and after the requisite 72-hour monitoring period he was discharged on 75 mg/day aspirin and 75 mg/day clopidogrel for one month, followed by lifelong 75 mg aspirin thereafter with prophylactic tranexamic acid during clopidogrel therapy.

## 3. Discussion

In the context of full-thickness myocardial infarction, prompt restoration of coronary perfusion has been shown to reduce morbidity and mortality significantly, with percutaneous coronary intervention being the more effective revascularisation method [5, 6]. The issue in those with known coagulopathies undergoing PCI is the increased risk of significant bleeding complications. Therefore, difficult management decisions have to be made as to whether to delay PCI and sacrifice door-to-balloon time for the sake of procedural safety or vice versa. In our case, we took a thorough history of the patient's haemophilia background, and based on his lack of previous major haemorrhage, it was felt that in his case the risk of bleeding as a result of the procedure would be outweighed by the benefit of swift revascularisation without performing additional haematological investigation.

No guidance exists as to what factor VIII level is deemed acceptable prior to PCI. The pertinent figure in those undergoing major surgery, according to the World Haemophilia Federation recommendations, is 80–100%. In several case reports of haemophilia A patients undergoing PCI, a preprocedure factor VIII level of >60% has arbitrarily been aimed for through factor VIII replacement, with success achieved in terms of preventing major intra- and postprocedure haemorrhage. Patients with mild haemophilia exhibit normalisation of factor VIII levels at times of pain or infection and it is likely that our patient's periprocedural VIII level also was significantly above baseline.

Few of these case reports describe patients with ST-segment elevation, however, and the concomitant pressure to perform PCI as soon as possible without time to check factor VIII levels. The decision to take him directly to the angiography lab without having factor VIII replacement or haematology consult was made purely on the basis of his history; his haemophilia was hitherto well controlled. The advantages of giving as little factor VIII as possible would be avoiding the risk of developing factor VIII inhibitor antibodies or emergent infection and minimising the risk of acute thrombosis (including stent thrombosis) due to excessive factor VIII or coronary vasospasm after DDAVP.

To improve procedural safety, we undertook angiography using a radial rather than femoral approach. Being a smaller and more superficial vessel, this allowed us to achieve haemostasis more easily, thus minimising the risk of major procedural bleeds (i.e., retroperitoneal haemorrhage and large groin haematomas) associated with femoral puncture. The recent RIFLE-STEACS study indeed demonstrated a bleeding complication risk of 12.2% in femoral access PCI compared to 7.8% for radial access [7].

Heparin was used as the sole procedural anticoagulant in our case. The current trend is to use the direct thrombin inhibitor bivalirudin for haemophiliac patients as it is associated with a lower rate of bleeding complications and does not require dose adjustments to be made according to the APTT [8]. However, unlike heparin, bivalirudin is not easily reversible and thus the use of heparin may offer greater control in patients with natural bleeding tendencies. Radial access in this case allowed for detailed postprocedural observation and allowed the treatment with tranexamic acid only.

Antiplatelet agents are required after stent insertion to prevent in-stent thrombosis. The current guidelines suggest one year of dual antiplatelet therapy with aspirin and clopidogrel for drug eluting stents and one month for bare metal stents. Since haemophilia A is not associated with any qualitative or quantitative platelet abnormality, antiplatelet agents should be given in accordance with these guidelines: indeed acute stent thrombosis has been reported in coagulopathic patients who have not received dual antiplatelet therapy after stenting [9]. Antiplatelet therapy does increase the risk of bleeding diathesis in patients with haemophilia A, and for that reason, a bare metal stent was chosen in order to facilitate the early withdrawal of clopidogrel. This approach is endorsed by the latest European Society of Cardiology guidelines, which advocate the use of bare metal stents in patients with haemophilia.

In summary, we describe the case of a middle-aged man with mild haemophilia A presenting with an acute anterior STEMI who was treated with primary PCI to his LAD. By taking a thorough history of his haemophilia, we were able to satisfactorily establish that he was at low risk of significant bleeding without having to determine a factor VIII level, thus optimising his door-to-balloon time. The procedure was carried out safely and with no other special considerations, save for the use of a bare metal stent to ensure minimum possible time on dual antiplatelet therapy. After 18 months of follow-up, he has not encountered any complications and has not required any factor VIII replacement.
